# Prevalence of Trachoma After Three Rounds of Antibiotic Mass Drug Administration in 13 Woredas of Gambella Region, Ethiopia

**DOI:** 10.1080/09286586.2023.2248624

**Published:** 2023-11-30

**Authors:** Addisu Alemayehu, Ademe Mekonen, Belete Mengistu, Addisalem Mihret, Aemiro Asmare, Ana Bakhtiari, Bekele Mengistu, Cristina Jimenez, Demis Kebede, Doul Bol, Fentahun Tadesse, Fikreab Kebede, Genet Gebru, Hannah Frawley, Jeremiah Ngondi, Mohammed Jemal, Molly Brady, Nebiyu Negussu, Robert Butcher, Scott McPherson, Sharone Backers, Anthony W. Solomon, Michael Dejene Bejiga, Emma M. Harding-Esch

**Affiliations:** a Act to End NTDs East, RTI International, Addis Ababa, Ethiopia End NTDs East, RTI International, Addis Ababa, Ethiopia; b Health Promotion and Disease Prevention Core Process, Gambella Regional Health Bureau, Ethiopia; c Task Force for Global Health, Atlanta, Georgia, USA; d Nekemte Specialized Hospital, Eastern Wollega Zone, Oromia Region, Ethiopia; e Sightsavers, Haywards Heath, UK; f Neglected Tropical Diseases Team, Disease Prevention and Control Directorate, Federal Ministry of Health, Addis Ababa, Ethiopia; g Act to End NTDs East, RTI International, Washington, DC, USA; h Clinical Research Department, London School of Hygiene & Tropical Medicine, London, UK; i Department of Control of Neglected Tropical Diseases, World Health Organization, Geneva, Switzerland; j Sightsavers, Addis Ababa, Ethiopia

**Keywords:** Elimination, ethiopia, gambella, neglected tropical diseases, prevalence, trachoma

## Abstract

**Background:**

Following baseline surveys in 2013 and 2014, trachoma elimination interventions, including three rounds of azithromycin mass drug administration (MDA), were implemented in 13 woredas (administrative districts) of Gambella Regional State, Ethiopia. We conducted impact surveys to determine if elimination thresholds have been met or if additional interventions are required.

**Methods:**

Cross-sectional population-based surveys were conducted in 13 woredas of Gambella Regional State, combined into five evaluation units (EUs), 6−12 months after their last MDA round. A two-stage systematic (first stage) and random (second stage) sampling technique was used. WHO-recommended protocols were implemented with the support of Tropical Data. Household water, sanitation and hygiene (WASH) access was assessed.

**Results:**

The age-adjusted prevalence of trachomatous inflammation—follicular (TF) in 1–9-year-olds in the five EUs ranged from 0.3–19.2%, representing a general decline in TF prevalence compared to baseline estimates. The age- and gender-adjusted prevalence of trachomatous trichiasis (TT) unknown to the health system in those aged ≥ 15 years ranged from 0.47–3.08%. Of households surveyed, 44% had access to an improved drinking water source within a 30-minute return journey of the house, but only 3% had access to an improved latrine.

**Conclusion:**

In two EUs, no further MDA should be delivered, and a surveillance survey should be conducted after two years without MDA. In one EU, one further round of MDA should be conducted followed by another impact survey. In two EUs, three further MDA rounds are required. Surgery, facial cleanliness and environmental improvement interventions are needed throughout the region.

## Introduction

Trachoma is an infectious disease caused by ocular infection with *Chlamydia trachomatis*. The infection is spread by direct and indirect contact with ocular and nasal discharges from infected people.^1–3^ Recurrent episodes of conjunctival infection can initiate a scarring process that, in severe cases, leads to in-turning of the eyelid, causing eyelashes to touch the eyeball, resulting in abrasion and ultimately irreversible blindness.^3,4^ Trachoma is common in Ethiopia, where about 65 million people are living in districts in need of treatment with antibiotics, facial cleanliness and environmental improvement for elimination of trachoma as a public health problem.^5^

The World Health Organization (WHO) recommends the SAFE (Surgery, Antibiotics, Facial cleanliness, and Environmental improvement) strategy to eliminate trachoma as a public health problem. The original target date was 2020,^6^ recently updated to 2030 in the neglected tropical disease (NTD) road map 2021–2030.^7^ The three criteria for achieving elimination are: (1) prevalence of trachomatous inflammation—follicular (TF) in 1–9-year-olds of < 5% in each formerly endemic district; (2) prevalence of trachomatous trichiasis (TT) unknown to the health system in those aged ≥15 years of < 0.2% in each formerly endemic district; (3) a system to identify and manage incident cases of TT.^8,9^

Ethiopia bears the highest burden of trachoma of any country worldwide.^5^ Gambella region is in the western part of Ethiopia, bordering Oromia to the North and the East, and the Southern Nations and Nationalities and People’s Regional state to the South. It also shares a border with South Sudan, where the prevalence of trachoma has been very high.^10,11^ In 2013 and 2014, baseline trachoma surveys were conducted in all 13 woredas (a woreda is a third-level administrative division of Ethiopia, after regional states and zones) of Gambella, surveyed as three evaluation units (EUs; defined by WHO as “the normal administrative unit for health care management, consisting of a population unit between 100,000–250,000 persons”).^12,13^ The baseline prevalence of TF in 1 − 9-year-olds was 11.5%, 12.5% and 19.3% for the three EUs, and a total of 142 cases of TT (3.8% of the surveyed ≥ 15-year-old population) were identified.^12^

Based on the baseline TF prevalence, three annual rounds of mass drug administration (MDA) of azithromycin (donated by Pfizer Inc., managed by the International Trachoma Initiative^14^) were implemented in all three EUs, covering all 13 endemic woredas of Gambella. No specific facial cleanliness and environmental improvement (the F and E of SAFE) activities were undertaken during this period. Here, we present the results of trachoma impact surveys undertaken 6 − 12 months after the last MDA round in these EUs to investigate whether the elimination thresholds have been met or if additional interventions are required.

## Methods

### Ethical approval and consent

Ethical clearance was obtained from the ethics committee of the Gambella Regional Health Bureau. Tropical Data support for trachoma surveys was approved by the London School of Hygiene & Tropical Medicine Observational Ethics Committee (16105). Zonal and Woreda Health officials approved the survey field work. Informed verbal consent was obtained from household heads and from each individual aged ≥15 years. For participants aged 1–14 years, informed verbal consent was obtained from a parent or guardian. Verbal consent for all individuals was recorded in the Tropical Data Android application.

### Study design and participant selection

The 13 woredas of Gambella were grouped into five EUs, based on the WHO EU definition for trachoma surveys.^13^ Baseline surveys in Gambella consisted of three EUs. These three EUs were split into five post-MDA EUs covering the same woredas, with a population ranging from 55,525 (combining Abobo and Gambella) to 123,635 (combining Akobo, Makuey, Wantuwa and Jikow). In Ethiopia, EUs are equivalent to woredas for the purposes for health care management, even though the population size may be below the minimum 100,000 population mentioned in the WHO EU definition. As per WHO recommendations, a two-stage cross-sectional survey design was employed.^15^ First, 26 villages (the primary sampling unit) were systematically selected from a list of all villages in each EU, using a probability proportional to size method. In the second stage, a compact segmentation method was employed to pick 30 households from the selected villages.^15^ Residents of those households aged ≥1 year were enumerated and invited to participate in the survey. To estimate a TF prevalence of 4% with a precision of ± 2%, we aimed to enumerate 1,164 children per EU on the basis this would yield 970 for examination, allowing for a non-response inflation factor of 1.2.

### Field team training

Eye care workers with previous community trachoma survey experience were recruited to train as trachoma graders. Graders were trained to identify TT, TF, trachomatous inflammation—intense (TI) and trachomatous scarring (TS) by Tropical Data-certified grader trainers using a standardised four-day programme of theoretical and practical training.^16^ Only grader trainees who scored a kappa score ≥ 0.7 for TF diagnosis in two inter-grader agreement (IGA) tests (one with 50 photos against consensus grading by five international experts; the other with 50 live participants, of whom at least five had TF, compared to a grader trainer), were deployed for the field work. Graders were trained to identify trichiasis, TI and TS using photographs of real cases, however field-based IGA testing was not used for these clinical signs because in most endemic areas, the prevalence is too low to allow for meaningful IGA testing, and furthermore, for TI and TS, there is no recommended programmatic response for high prevalences of these signs.^17^ The recorder trainees had both a diploma and first degree in health and technology-related fields and only those who passed a recorder assessment joined survey teams. A total of 13 survey teams, each consisting of one grader and one recorder, were subsequently formed and deployed to the field; the 13 teams surveyed each EU one by one. Each team had a driver and was accompanied by a local village guide. A senior ophthalmologist supervised the team during the surveys. Surveys started in February 2019 and finished in March 2019.

### Household- and individual-level data collection

Teams moved house-to-house during the surveys. Upon arrival at each household, the household head or their nominee was asked a United Nations Children’s Fund (UNICEF)/WHO Joint Monitoring Programme (JMP) household questionnaire modified for trachoma surveys to assess the availability and status of household water, sanitation and hygiene (WASH) access.^16–18^

All consenting residents were examined for clinical signs of trachoma using the WHO simplified grading system.^19^ Grading was undertaken in natural or torch light under 2.5× magnification, and the presence or absence of TT, TF and TI in each examined eye recorded. To aid with the diagnosis of TF, follicle size guides were used.^20^ When TT was detected, the individual was asked whether a health worker had previously offered them surgery or epilation, and the presence or absence of TS in that eye was also assessed. Graders used disposable gloves or cleaned their hands with 70% alcohol after each examination to avoid spread of infection between participants. All individuals with active (inflammatory) trachoma were provided with two tubes of 1% tetracycline eye ointment free of charge to apply twice daily for 6 weeks. Participants with significant ocular pathology, including trichiasis, were referred to appropriate services. We employed standard quality control and quality assurance measures for trachoma prevalence surveys.^21^

### Data analysis

Data were collected electronically using the Tropical Data collection tool,^22^ an Open Data Kit-based application running on Android smartphones. Data were encrypted and securely transferred to a central server managed by Tropical Data, where they were checked in near real time. At the end of the survey, prevalence estimates were generated using the analysis methods developed within the Global Trachoma Mapping Project (GTMP), which included adjustment for the age (and, for TT, gender) distribution of the underlying population using the most recent census.^17^

Comparisons of impact survey data with GTMP baseline data were made at face value and were not evaluated with statistical tests because the EU boundaries changed between the baseline and impact survey series. The baseline prevalence of each impact survey EU was presumed to be the prevalence of the geographically overlapping baseline EU.

The association between trachoma and individual and household-level variables was assessed using a mixed-effects regression model using the statistical software R^23^ and the package lme4,^24^ with village (TT models) or village and household (TF models) of residence as random effects parameters. Variables were first tested in univariable analysis. Univariable models were compared to null models with likelihood ratio tests. Those with a significant (*p* < .05) association in univariable analysis were included in the multivariable model if they were not co-linear with other variables. Other potential variables for the multivariable model were tested in a step-wise manner and included if they improved model fit.

## Results

### Surveyed population

Data collection for this series of impact surveys took place in February – March 2019. Across the five EUs, a total of 14,904 individuals were enumerated and 14,013 examined. Among the examined study population, 8,155 (58%) were female. A total of 678 individuals were absent on the day of the household visit and 212 refused to participate in the study. The numbers enumerated and examined in each EU are shown in Table 1.

### Clinical signs of trachoma

**Figure 1:**
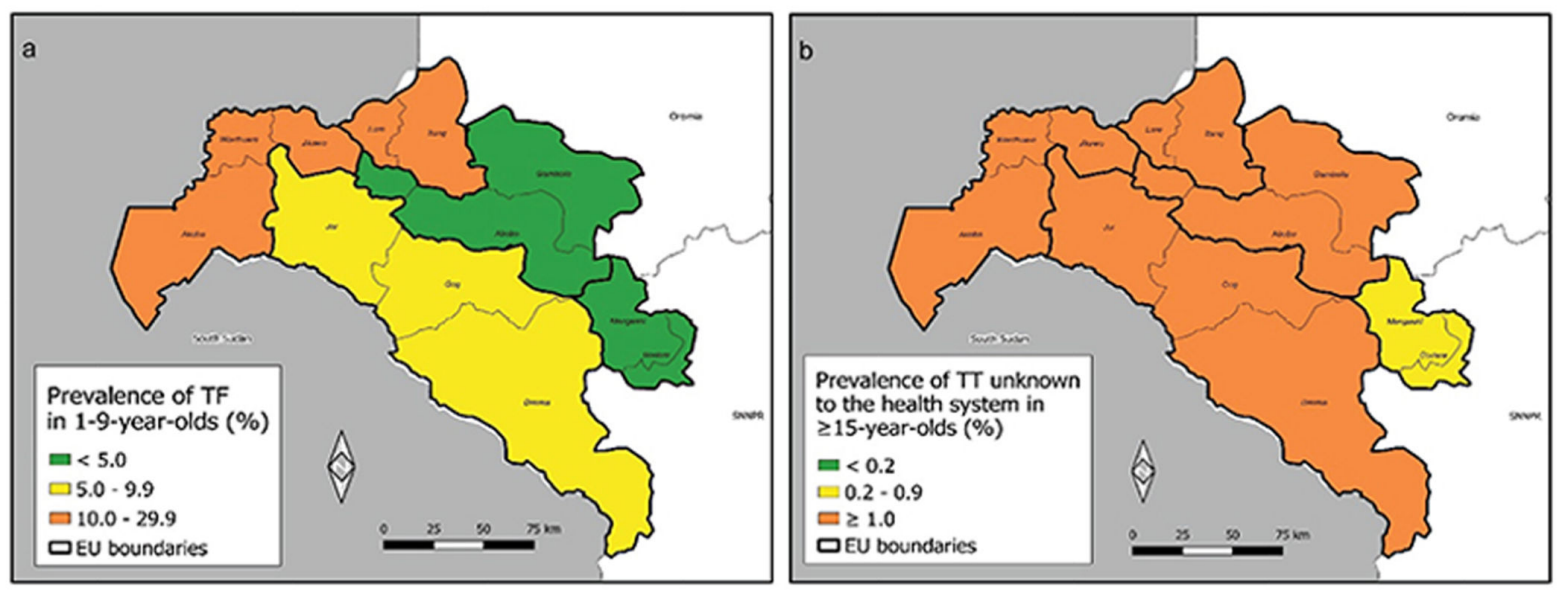
(a) Age-adjusted prevalence of trachomatous inflammation—follicular (TF) in 1–9-year-olds and (b) age- and gender-adjusted prevalence of trachomatous trichiasis (TT) unknown to the health system in ≥ 15-year-olds in trachoma impact surveys, Gambella, Ethiopia, February–March 2019. The boundaries and names shown and the designations used on this map do not imply the expression of any opinion whatsoever on the part of the authors, or the institutions with which they are affiliated, concerning the legal status of any country, territory, city or area or of its authorities, or concerning the delimitation of its frontiers or boundaries. Dotted lines on maps represent approximate border lines for which there may not yet be full agreement. EU: Evaluation Unit.

**Figure 2:**
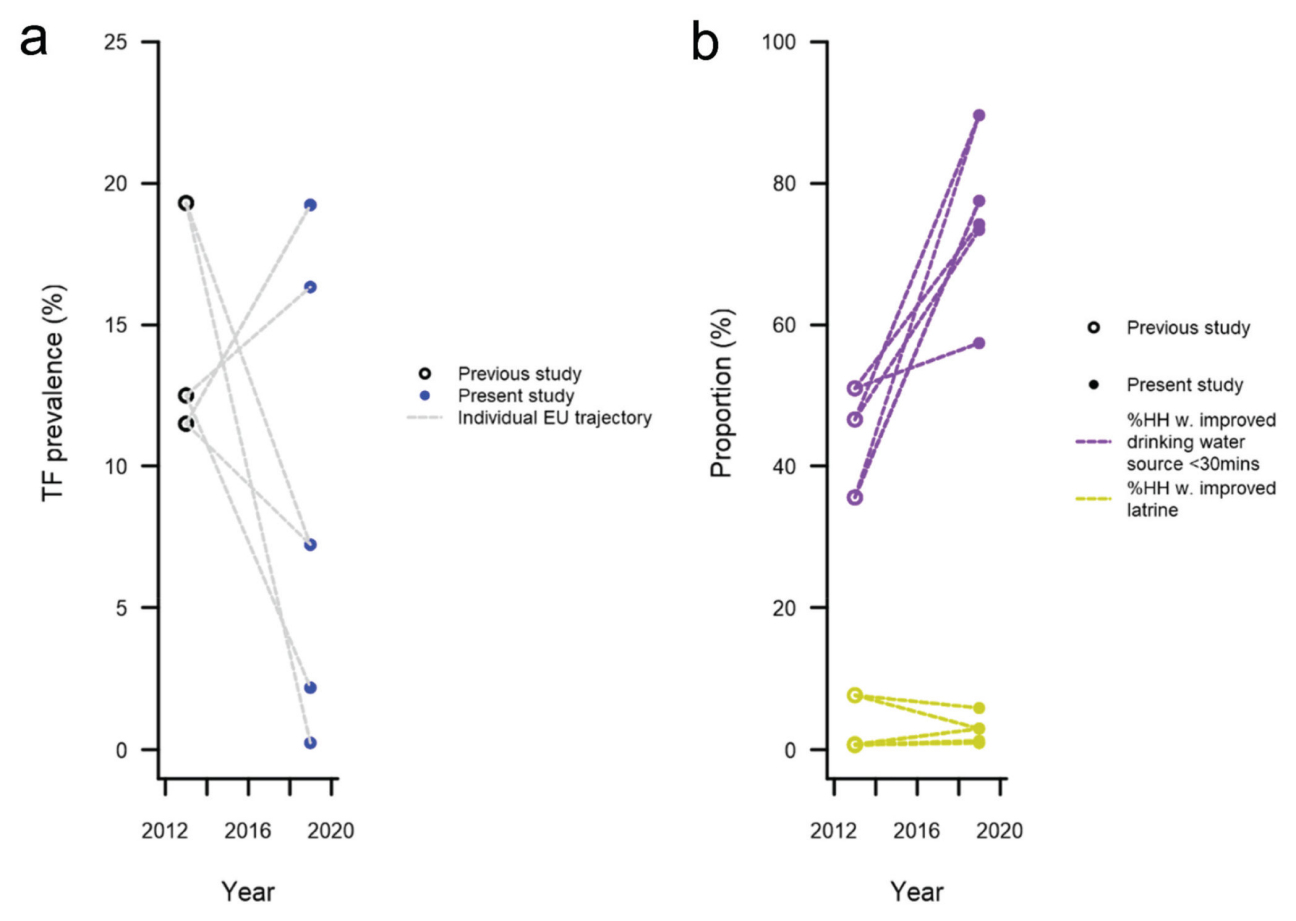
(a) Change in prevalence of trachomatous inflammation—follicular (TF) between baseline and impact surveys in Gambella region, Ethiopia. (b) Change in proportion of households with an improved drinking water source within a 30-minute return journey of the house, and proportion of households with an improved latrine between baseline and impact surveys, Gambella region, Ethiopia. The baseline survey data have been published elsewhere.^12^ No statistical analysis of comparison is provided because the EU boundaries were not the same between pre- (baseline) and post-MDA (impact) surveys.

Of 14,013 people examined, 5,489 were aged 1–9 years. 594 of those children had TF in at least one eye. The lowest prevalence of TF in 1–9-year-olds was 0.3% (95% confidence interval [CI]: 0.0–0.7%) in the EU covering Mengeshi and Godere woredas. The highest prevalence of TF was 19.2% (95% CI: 14.4–24.1%) in the EU covering Wanthuwa, Makoy, Jikawo and Akobo woredas. The prevalence of TF in children was < 5% in two EUs, 5.0–9.9% in one EU and ≥ 10% in two EUs (Figure 1). The number of children with TF and EU-level prevalences are shown in Table 2. The prevalence of TF changed since the baseline surveys (Figure 2a), with three impact survey EUs having lower prevalence compared with their baseline EU, and two having higher prevalence.

Among 6,942 adults aged ≥15 years examined, 265 (4%) were found to have TT (Table 3). 189/265 (71%) of those with TT also had TS in the eye with TT. 243/265 (92%) of the people with TT reported not being offered management and were classed as “unknown to the health system”. The lowest age- and gender-adjusted prevalence of TT unknown to the health system in those aged ≥15 years was 0.47% (95% CI: 0.20–0.85%), reported in the EU covering Godere and Mengeshi woredas in Mejang zone. The highest age- and gender-adjusted prevalence of TT unknown to the health system in those aged ≥15 years was 3.08% (95% CI: 2.14–4.20%), reported in the EU covering Wanthuwa, Makoy, Jikawo and Akobo woredas in Nuer zone. The age- and gender-adjusted prevalence of TT unknown to the health system in those aged ≥15 years was between 0.2–0.9% in one EU, and ≥ 1.0% in four EUs (Figure 1).

### Household level water, sanitation and hygiene access

The outcomes of the household WASH questionnaire are shown in Table 4. Of 3,880 households surveyed, 1,706 (44%) reported having an improved drinking water source within a 30-minute return journey of the house. This ranged from 39% in the EU covering Gog, Jor and Dima woredas to 51% in the EU covering Mengeshi and Godere woredas. However, only 110 (3%) households surveyed reported having access to an improved latrine (range: 1–6%). 101 (3%) households had a hand-wash station within 15 metres of their latrine. Compared to baseline data, the proportion of households with an improved drinking water source within a 30-minute return journey had increased in all impact surveys EUs. The proportion of households with an improved latrine was similar to baseline estimates (Figure 2b).

### Association between clinical signs of disease and individual- and household-level variables

Amongst 1–9-year-olds examined, there was strong evidence that TF was less common in older children than younger children (compared to 1–3-year-olds, the adjusted odds ratio [aOR] for TF in 4–6-year-olds was 0.7 [95% CI: 0.5–0.8] and for 7–9-year-olds was 0.2 [95% CI: 0.1–0.2]; *p* < .001). TF was more common in children living in households practising open defecation (aOR: 3.1 [95% CI: 1.8–5.3]; *p* < .001) compared to households not practising open defecation, and in children living in households using a surface water source for their washing water compared with those not using surface water (aOR: 2.0 [95% CI: 1.3–3.2]; *p* = .003). There was marginal evidence that TF was less common in female children than in male children (aOR: 0.8 [95% CI: 0.7–1.0]; *p* = .050) (Supplementary table S1).

TT was more common in older people (compared to 15–34-year-olds, the aOR for TT in 35–54-year-olds was 4.4 [95% CI: 3.2–6.0], for 55–74-year-olds was 9.1 [95% CI: 6.3–13.2] and for ≥ 75-year-olds was 10.8 [95% CI: 4.9–23.7]; *p* < .001). TT was more common in female ≥ 15-year-olds (aOR: 3.0 [95% CI: 2.2–4.2]; *p* < .001) than males in this age bracket (Supplementary table S2).

## Discussion

The trachoma impact survey results reported here present a mixed picture for Gambella region. The prevalence of TF in 1–9-year-olds was < 5% following three rounds of MDA in two of the five EUs surveyed,5.0–9.9% in one EU and remained ≥ 10% in two EUs. The prevalence of TT unknown to the health system in ≥ 15-year-olds was ≥ 0.2% in all five EUs. There are clear short-term programmatic implications from these findings guided by WHO recommendations.^3,25,26^ First, in the two EUs with a TF prevalence < 5%, no further MDA should be conducted for two years, at which time another prevalence survey should be carried out (referred to as a surveillance survey). One and three rounds of MDA should be carried out in the EUs with TF prevalence estimates of 5.0–9.9% and ≥ 10%, respectively, before another impact survey is undertaken. Active TT case finding campaigns and strengthening of surgical services has been implemented since 2019 in partnership with the African Medical and Research Foundation (AMREF) and should continue throughout the region to address the TT burden. We demonstrate in this manuscript that, as found in other trachomaendemic settings,^27,28^ TT is more common in older people and in women, so programmes must be designed to ensure these groups are not excluded when implementing interventions.

According to the 2016 Ethiopia Demographic and Health Survey (EDHS),^29^ the sustainability of water schemes and the non-functionality of water supply are a challenge in Gambella region. To date, there has been no external partner support and therefore no funding available for programmatic implementation of F and E activities in these survey regions. However, consecutive EDHSs between 2011 and 2016 have shown an increase in households using an improved water source for their drinking water (from 68 to 84%) and a decrease in the number of households needing to travel more than 30 minutes to their drinking water source (from 22 to 13%). One of the major tasks for the regional Water and Energy bureau in Gambella is to increase access for improved drinking water to the people living in the region. Three governmental bureaus (Water and Energy, Education, Health) have collaborated on WASH programmes and infrastructure construction to improve drinking water access. In addition to this, even though there were no formal partners directly working on WASH, there was some support from UNICEF for WASH programmes in the region. It is therefore likely that both governmental and UNICEF action are responsible for some of the considerable improvement in the proportion of households with improved drinking water from the baseline survey. In this survey series, 44% of households had an improved drinking water source within a 30-minute round trip of the house, which is broadly comparable to the 2016 estimates. The trend observed between the pre- and post-MDA surveys also suggested an improvement in recent years. However, there remained an appreciable number of households using surface water sources for washing water collection; and children living in these households were more likely to have active trachoma. The EDHS’s between 2011 and 2016 showed a decrease in the number of households using an improved latrine (from 12 to 8%). Furthermore, a knowledge, attitudes and practices study conducted by UNICEF in eight regions of Ethiopia in 2017 reported that families who have toilets practise open defecation at times when their toilets are not fully functional. Unavailability of public latrines aggravates open defecation, and in areas where they are available, poor maintenance discourages people from using them.^30^ The estimates of improved latrine access in this survey series were still lower at 3% of households.^29^ This persistently low level is a clear sign of the continued problems of open defecation in the region and the need to provide improved latrines that will be maintained and used. Other stakeholders have previously advocated for these improvements.^31^ Promotion of F and E should be implemented throughout the region. There is a particularly urgent and widespread need for general improvement in WASH facilities in Gambella. These improvements are central to the recently released roadmap 2021–2030 for the sustainable elimination of NTDs,^7^ and are likely to also have significant benefits beyond trachoma elimination.^7^

These data highlight some outstanding challenges for the trachoma programme in Gambella. First, the TT prevalence was high and the majority of cases (>90%) reported not having previously been offered management. Incident cases of TT will need to be managed for many years after the prevalence of TF in children has been reduced, so there will be an ongoing need to establish mechanisms to identify and manage cases. Integration of TT services into routine health care will be important to ensure sustainability of these services. Second, it is concerning that TF continued to exceed the elimination threshold in some EUs, despite the delivery of three rounds of MDA. The success of MDA programmes has been variable throughout Ethiopia^32–34^ and operational research is sorely needed to understand the reasons for this.^35^ It is important to understand how well the programme has reached its operational coverage targets across the three control activities for active trachoma (the A, F and E elements of the SAFE strategy). Average MDA coverage in the 13 woredas was 92% in 2016, 96% in 2017 and 89% in 2018. Over all three treatment points, only three woredas had a program coverage below the minimum WHO-recommended coverage of 80%,^13^ one woreda in 2016 (52%, Dimma) and two in 2018 (66%, Gog and 73%, Godere). This would suggest that the implementation of MDA in these regions has been generally successful in terms of coverage, and therefore it may be valuable to better understand the prevalence, intensity and transmission dynamics of ocular *C. trachomatis* infection in the region to determine additional areas which could be targeted in future interventions. Future research on these topics should account for the fact that sociocultural practices vary widely between zones, so combining woredas from multiple zones into single EUs may fail to capture variation in behaviours and transmission dynamics relevant to F and E components of the SAFE strategy. Gambella region faces the additional challenge of immigration of displaced populations from the ongoing conflict in South Sudan, which could introduce new infections into treated communities.^10^ Refugee settlements should continue to be included in future surveillance activities.

There were limitations to this study. The surveys were designed to detect TF prevalence of 4%, with an absolute precision of 2%. Despite reaching within ten of the targeted number households in all EUs, the sample size to estimate TF prevalence with this level of precision (970 children) was not achieved in two of the five EUs. Both EUs had a TF prevalence < 5% and, despite the confidence interval being wider than planned, its upper boundary remained below 5%. The sample size was also insufficient to accurately measure TT prevalence in all EUs surveyed. TT survey guidelines from WHO suggest at least 2,818 adults or 30 clusters should be surveyed to accurately estimate TT prevalence.^36^ As neither target was met in any EU of this survey series, we acknowledge the insufficiency of the sample to accurately estimate TT prevalence. The resulting estimates should therefore be treated with caution, and the presented confidence intervals should be taken into account when interpreting the data. We note that 71% of people with TT had TS in the eye with TT. We acknowledge that the Tropical Data grading system dedicates very little time to teaching about TS and does not formally assess agreement between graders in the grading of TS, therefore this estimate is subject to inaccuracies due to potential inconsistencies in grading. Finally, the definition of TT has recently been updated to require that deviated eyelashes from the upper eyelid only be graded as TT.^37,38^ During this study, which was conducted before the change in TT definition was published, data were not collected on the eyelid of origin of deviated eyelashes, so caution should be applied when comparing the data presented here to later estimates.

## Conclusions

Following region-wide scale-up of trachoma elimination activities in Gambella region of Ethiopia, some progress has been made towards reaching the elimination targets but there is still much work to do. Ongoing intervention with the full SAFE strategy is required in three of the five EUs surveyed in Gambella region, and the S, F and E components should also continue in the remaining two EUs. There remain programmatic challenges in this region, which would benefit from operational research.

## Supplementary Material

Supplemental data for this article can be accessed online at https://doi.org/10.1080/09286586.2023.2248624

Supplemental material

## Figures and Tables

**Table 1:** Population aged ≥1 year participating in trachoma impact surveys, Gambella Region, Ethiopia, February-March 2019.

Zone	Woredas forming evaluation unit	Evaluation Unit ID	Total population Enumerated (n)	Total population absent (n)	Total population refused (n)	Total population examined (n, %)	Examined female (n, %)
Agnua II	Jor, Gog and Dimma	80965	3,009	144	89	2,776 (92)	1,659 (60)
	Gambella and Abobo	80966	2,800	191	100	2,508 (90)	1,523 (61)
Nuer II and Itang	Lare and Itang	80967	2,995	116	17	2,862 (96)	1,701 (59)
Mejang	Mengeshi and Godere	80968	3,048	150	0	2,898 (95)	1,587 (55)
Nuer II	Wanthuwa, Makoy, Jikawo and Akobo	80969	3,052	77	6	2,969 (97)	1,685 (57)
*Total*	*-*	-	*14,904*	*678*	*212*	*14,013 (94)*	*8,155 (58)*

**Table 2:** Proportion of children aged 1–9 years with trachomatous inflammation—follicular (TF) and trachomatous inflammation—intense (TI) in trachoma impact surveys, Gambella Region, Ethiopia, February–March 2019.

Zone	Evaluation Unit	Total children examined (n)	Children with TF (n, %)	Children with TI (n, %)	Age-adjusted prevalence of TF in 1–9-year-olds (%, 95% CI)
Agnua II	Jor, Gog and Dimma	1,035	82 (8)	5 (<1)	7.2 (3.8–11.4)
	Gambella and Abobo	867	21 (2)	2 (<1)	2.2 (1.2–3.3)
Nuer II and Itang	Lare and Itang	1,247	224 (18)	56 (4)	16.3 (11.9–20.9)
Mejang	Mengeshi and Godere	821	2 (<1)	0 (0)	0.3 (0.0–0.7)
Nuer II	Wanthuwa, Makoy, Jikawo and Akobo	1,519	265 (17)	49 (3)	19.2 (14.4–24.1)
**Total**		5,489	594 (11)	112 (2)	-

**Table 3:** Proportion of adults aged ≥15 years with trachomatous trichiasis (TT) in trachoma impact surveys, Gambella Region, Ethiopia, February–March 2019.

Zone	Woredas in evaluation unit	Number of ≥ 15-year-olds examined (n)	Cases of TT in ≥ 15-year- olds (n, %)	Cases of TT and TS in ≥ 15-year- olds (n, %)	Cases of TT unknown to the health system in ≥ 15- year- olds (n, %)	Age- and gender-adjusted prevalence of TT unknown to the health system in ≥ 15-year-olds (%, 95% CI)
Agnua II	Jor, Gog and Dimma	1,372	47 (3)	19 (1)	47 (3)	1.72 (1.05–2.56)
	Gambella and Abobo	1,307	34 (3)	6 (<1)	30 (2)	1.31 (0.67–2.13)
Nuer II and Itang	Lare and Itang	1,316	71 (5)	63 (5)	65 (5)	2.72 (1.77–3.84)
Mejang	Mengeshi and Godere	1,742	17 (1)	8 (<1)	15 (1)	0.47 (0.20–0.85)
Nuer II	Wanthuwa, Makoy, Jikawo and Akobo	1,205	96 (8)	93 (8)	86 (7)	3.08 (2.14–4.20)
Total		6,942	265 (4)	189 (3)	243 (4)	-

CI: confidence interval; TT: trachomatous trichiasis (defined as at least one eyelash rubbing on the eyeball or evidence of recent removal of inturned eyelashes)^19;^ TS : trachomatous scarring.

**Table 4:** Coverage of water, sanitation and hygiene facilities of households in trachoma impact surveys, Gambella Region, Ethiopia, February-March 2019.

Zone	Evaluation unit	Households surveyed (n)	Households with improved drinking water source within 30-minute return journey (n, %)*	Households with an improved latrine (n, %)	Households with a latrine with a handwashing station (n, %)
Agnual II	Jor, Gog and Dimma	777	302 (39)	23 (3)	48 (6)
Agnua II	Gambella and Abobo	775	334 (43)	10 (1)	3 (<1)
Nuer II and Itang	Lare and Itang	774	315 (41)	23 (3)	0 (0)
Mejang	Mengeshi and Godere	779	400 (51)	46 (6)	49 (6)
Nuer II	Wanthuwa, Makoy, Jikawo and Akobo	775	355 (46)	8 (1)	1 (<1)
Total		3,880	1,706 (44)	110 (3)	101 (3)

* Households without access to an improved water source were by default defined as being in the “over 30 minutes” category.
