# Supplementary material for: Prevalence of Trachoma After Three Rounds of Antibiotic Mass Drug Administration in 13 Woredas of Gambella Region, Ethiopia
Source: Ophthalmic Epidemiol. Author manuscript; Available in PMC 2026 Mar 9. (PMC7618835; doi:10.1080/09286586.2023.2248624)
Supplement: Supplemental material [file EMS212684-supplement-Supplemental_material.zip › iope_a_2248624_sm0937.docx]

**Prevalence of trachoma after three rounds of antibiotic mass drug administration in 13 woredas of Gambella Region, Ethiopia**

Addisu Alemayehu^1^, Ademe Mekonen^2^, Belete Mengistu^1^, Addisalem Mihret^1^, Aemiro Asmare^1^, Ana Bakhtiari^3^, Bekele Mengistu^4^, Cristina Jimenez^5^, Demis Kebede^1^, Doul Bol^2^, Fentahun Tadesse^6^, Fikreab Kebede^6^, Genet Gebru^6^, Hannah Frawley^7^, Jeremiah Ngondi^7^, Mohammed Jemal^2^, Molly Brady^7^, Nebiyu Nigussu^6^, Robert Butcher^8^, Scott McPherson^7^, Sharone Backers^1^, Anthony W. Solomon^9^, Michael Dejene^10^, Emma M. Harding-Esch^8^.

**Supplementary tables**

**Supplementary table 1.** Univariable and multivariable binomial logistic regression outcomes for association between trachomatous inflammation—follicular (TF), age, gender and household-level water access and sanitation variables. All models include household and cluster of residence as random effects variables. Odds ratio confidence intervals generated using Wald’s method. Significance tested using likelihood ratio tests. 14 children lived in households where the facilities could not be categorized with the options available on the data collection form (questionnaire response was ‘Other’), so were removed from the analysis. These regression analyses were therefore carried out on 5,475 children.

| **Variable** | **Level** | **No TF** | **TF** | **Univariable** | | **Multivariable** | |
| --- | --- | --- | --- | --- | --- | --- | --- |
|  |  |  |  | **OR (95% CI)** | **p** | **aOR (95% CI)** | **p** |
| Age group (years) | 1–3 | 1,583 | 312 | Reference | <0.001 | Reference | <0.001 |
|  | 4–6 | 1,655 | 221 | 0.7 (0.5–0.8) |  | 0.7 (0.5–0.8) |  |
|  | 7–9 | 1,649 | 55 | 0.2 (0.1–0.2) |  | 0.2 (0.1–0.2) |  |
| Gender | Male | 2,421 | 330 | Reference | 0.007 | Reference | 0.050 |
|  | Female | 2,466 | 258 | 0.8 (0.6–0.9) |  | 0.8 (0.7–1.0) |  |
| Number of household residents aged 1–9 years | 1–2 | 2,592 | 280 | Reference | 0.680 | Not tested | |
|  | 3–4 | 1,853 | 247 | 1.1 (0.9–1.4) |  |  |  |
|  | ≥5 | 442 | 61 | 1.1 (0.8–1.7) |  |  |  |
| Open defecation | No | 1,254 | 32 | Reference | <0.001 | Reference | <0.001 |
|  | Yes | 3,633 | 556 | 3.2 (1.9–5.4) |  | 3.1 (1.8–5.3) |  |
| Time taken for return journey to washing water source | <30 minutes | 3,354 | 466 | Reference | 0.289 | Not tested | |
|  | ≥30 minutes | 1,533 | 122 | 1.2 (0.8–1.9) |  |  |  |
| Surface washing water source | No | 3,825 | 407 | Reference | 0.001 | Reference | 0.003 |
|  | Yes | 1,062 | 181 | 2.2 (1.4–3.5) |  | 2.0 (1.3–3.2) |  |
| aOR: adjusted odds ratio; CI: confidence interval; OR: odds ratio; TF: trachomatous inflammation—follicular. | | | | | | | |

**Supplementary table 2.** Univariable and multivariable binomial logistic regression outcomes for association between trachomatous trichiasis (TT; management status not specified), age and gender. All models include cluster of residence as a random effect variable. Odds ratio confidence intervals generated using Wald’s method. Significance tested using likelihood ratio tests. These regression models were run on all 6,942 ≥15-year-olds examined.

| **Variable** | **Level** | **No TT** | **TT** | **Univariable** | | **Multivariable** | |
| --- | --- | --- | --- | --- | --- | --- | --- |
|  |  |  |  | **OR (95% CI)** | **p** | **aOR (95% CI)** | **P** |
| Age group (years) | 15–34 | 4,487 | 79 | Reference | <0.001 | Reference | <0.001 |
|  | 35–54 | 1,671 | 112 | 3.9 (2.9–5.3) |  | 4.4 (3.2–6.0) |  |
|  | 55–74 | 451 | 65 | 7.7 (5.4–11.1) |  | 9.1 (6.3–13.2) |  |
|  | ≥75 | 68 | 9 | 8.0 (3.7–17.3) |  | 10.8 (4.9–23.7) |  |
| Gender | Male | 2,310 | 47 | Reference | <0.001 | Reference | <0.001 |
|  | Female | 4,367 | 218 | 2.2 (1.6–3.1) |  | 3.0 (2.2–4.2) |  |
| aOR: adjusted odds ratio; CI: confidence interval; OR: odds ratio; TT: trachomatous trichiasis. | | | | | | | |
